# Changes in Parameters after High Tibial Osteotomy: Comparison of EOS System and Computed Tomographic Analysis

**DOI:** 10.3390/jcm12175638

**Published:** 2023-08-29

**Authors:** Hyun-Jin Yoo, Jae-Kyu Choi, Youn-Moo Heo, Sung-Jun Moon, Byung-Hak Oh

**Affiliations:** Department of Orthopedic Surgery, College of Medicine, Konyang University, 158 Gwanjeodong-ro, Seo-gu, Daejeon 35365, Republic of Korea; yoo15love@gmail.com (H.-J.Y.); 400852@kyuh.ac.kr (J.-K.C.); hurym1973@hanmail.net (Y.-M.H.); 400911@kyuh.ac.kr (S.-J.M.)

**Keywords:** EOS imaging, computed tomography, rotational alignment, osteotomy, malalignment

## Abstract

Unintended rotation of the distal tibia occurs during medial open-wedge high tibial osteotomy (MOWHTO). Computed tomography (CT) is the standard method of measuring lower limb alignment; however, the new low-dose EOS system allows three-dimensional limb modeling with automated measurements of lower limb alignment. This study investigated the differences between the changes in lower limb alignment profiles obtained using the EOS system and CT in patients who underwent MOWHTO. We investigated whether any factors contributed to the degree of deformation. Thirty patients were prospectively enrolled between October 2019 and February 2023. Changes in femoral and tibial torsion, femorotibial rotation, and posterior tibial slope were measured using pre- and post-MOWHTO CT and EOS images. We found no significant difference in pre- and postoperative tibial torsion or posterior tibial slope between CT and EOS. No variables showed a significant correlation with changes in the tibial torsion or posterior tibial slope. This study confirmed the possibility that the EOS system could replace CT in measuring changes in several parameters pre- and postoperatively. Furthermore, we confirmed that the distal tibia tended to be internally rotated after MOWHTO; however, we found no significantly related parameters related to deformation caused by MOWHTO.

## 1. Introduction

Medial open-wedge high tibial osteotomy (MOWHTO) is a surgical technique widely performed in relatively young and active adults with medial compartment osteoarthritis, varus deformities of the knee joints, or anterior cruciate ligament deficiency [1,2,3,4,5]. This surgical procedure transfers mechanical loading from the damaged medial compartment to the intact lateral compartments. In addition, MOWHTO can delay or prevent deterioration of the medial compartment of the knee joint, thereby delaying the need for knee joint arthroplasty. Several studies have reported relatively satisfactory short- and mid-term results of this surgical method [6,7,8].

Accurate alignment correction in MOWHTO is essential for achieving satisfactory clinical outcomes. Surgeons performing MOWHTO have focused on correcting lower limb alignment in the coronal plane [9]. However, when MOWHTO is performed, three-dimensional (3D) structural deformation occurs in the coronal, axial, and sagittal planes [10]. Various studies have reported that MOWHTO causes unintended rotation of the distal tibia and alteration of the posterior tibial slope [11,12,13,14]. MOWHTO can also adversely affect the ankle and knee joints, causing patellofemoral arthritis, pes planus, and gait abnormalities [15,16,17].

Therefore, many studies on alignment changes after MOWHTO have been conducted and various discussions have been made on how to measure alignment of lower extremities as interest in the alignment of lower limbs before and after HTO has increased [10,14,18,19,20,21,22,23]. Various methods including clinical, sonographic, fluoroscopic, and magnetic resonance imaging have been proposed to measure the rotation of lower extremities, but none of them have been widely used routinely [24,25,26,27]. In recent years, computed tomography (CT) has become the gold-standard radiographic method for evaluating alignment [9,28].

However, a biplanar low-dose EOS system (EOS Imaging, Paris, France) has recently been developed to analyze lower limb alignment [29]. The EOS system enables significantly lower radiation exposure than conventional radiographs. Simultaneously, the EOS system captures whole-body anteroposterior (AP) and lateral two-dimensional radiographs in a scaled environment, allowing 3D reconstruction of the bone structures of the spine and lower extremities using stereoscopic radiography [30]. Thus, various clinical parameters of the lower extremities and spine, including the femoral and tibial torsions, can be measured using the 3D model [31,32].

Some studies have reported that the rotation of the lower extremities can be evaluated using the EOS system; however, few studies have investigated the use of the EOS system in assessing other factors, such as rotation after MOWHTO [33,34]. Therefore, we aimed to investigate the differences between the changes in lower limb alignment and rotation profiles obtained using the EOS system and CT in patients who underwent MOWHTO. We hypothesized that there would be no differences in the changes in lower limb alignment and rotation profiles between the EOS system and CT. Furthermore, we investigated whether other factors contributed to the amount of rotational deformation and slope changes.

## 2. Materials and Methods

### 2.1. Patients

**Figure 1:**
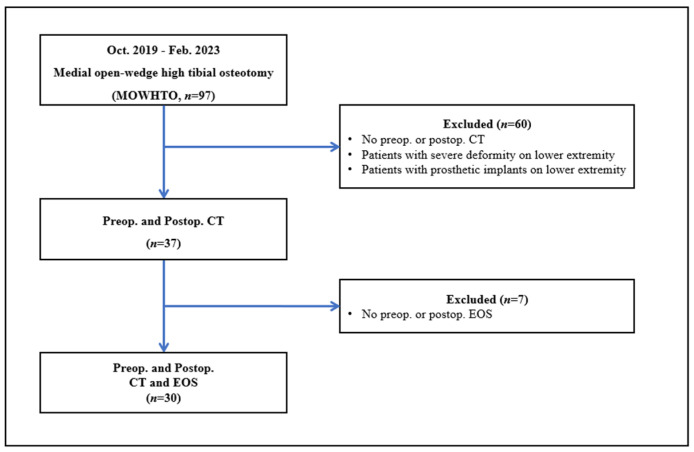
Flowchart of the study.

This prospective study was approved by the Institutional Review Board (IRB no. 2023-02-012). We prospectively included all patients who underwent MOWHTO between October 2019 and February 2023. Patients with severe lower extremity deformities such as traumas, fractures, or prosthetic implants in the lower extremity were excluded. In total, 97 patients (97 knees) met the criteria; however, 60 patients (60 knees) did not undergo pre- or postoperative CT. Furthermore, seven patients (seven knees) did not undergo EOS imaging studies as part of the preoperative work-up or to evaluate changes in lower limb alignment postoperatively. A flowchart of this study is shown in Figure 1.

### 2.2. Evaluation Methods

**Figure 2:**
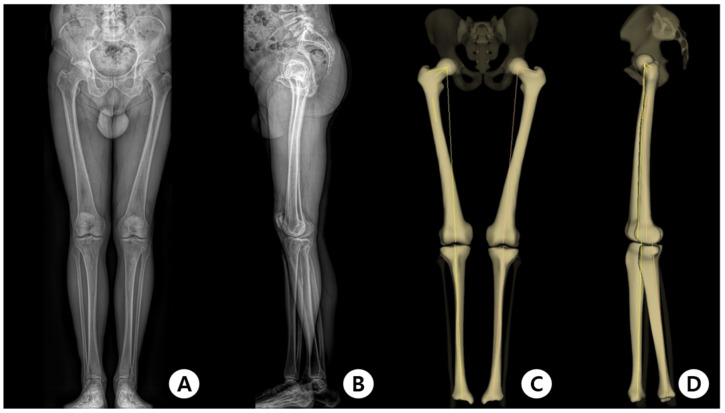
(**A**,**B**) Anteroposterior and lateral radiographs acquired for 3D reconstruction; (**C**,**D**) The 3D reconstruction images made by the EOS imaging system.

During the scan, the patients were instructed to touch both hands to their cheeks while holding their breath. We obtained standing AP and lateral images, including the entire lower extremity, and created images using the EOS imaging system. The images were stored on an institutional picture archiving and communication system (PACS) network. Subsequently, anatomical landmark identification was performed manually on the digital AP and lateral EOS two-dimensional radiographs using PACS workstation software tools (sterEOS; Biospace Med, Paris, France) (Figure 2A,B) [35]. Three-dimensional images were reconstructed by radiology technicians with several years of experience in musculoskeletal radiology who were trained in using sterEOS software. Various parameters of the lower extremities were calculated using these images by the computer-aided program (Figure 2C,D).

In addition, a helical CT machine (Aquilion Prime, Canon Medical Systems, Otawara, Japan) was used to obtain axial images of lower extremities with a voltage source of 120 kV, and slices were acquired at 3 mm intervals. The CT scan captured the whole lower extremity, and the threshold was defined from −1000 HU to 1000 HU to highlight the bone. Patients were instructed to straighten their legs while lying down, and radiologists fixed their legs with a device to prevent them from moving while undergoing CT. Furthermore, a simple radiographic evaluation was performed using weight-bearing lower extremity AP radiographs.

Measurements were performed using our institution’s PACS workstation (M6, INFINITT Healthcare Co., Ltd., Seoul, Republic of Korea). These were conducted by two senior orthopedists who were mainly involved in the care of lower extremities, including the hip and knee joints.

### 2.3. Measurement of Parameters and Clinical Outcomes

The femoral neck anteversion was computed as the angle between the femoral neck axis and the axis tangential to the posterior condyles on the transverse femoral plane [36]. The angle was positive when the femoral neck was anteverted. Moreover, the axis adapted to the posterior tibial plateau rim and the bimalleolar axis was defined as tibial torsion [37,38]. Femorotibial rotation was the angle between the axis of the posterior condyles of the femur and the posterior tibial plateau rim. The values were positive when the distal fragment externally rotated during tibial torsion and femorotibial rotation.

**Figure 3:**
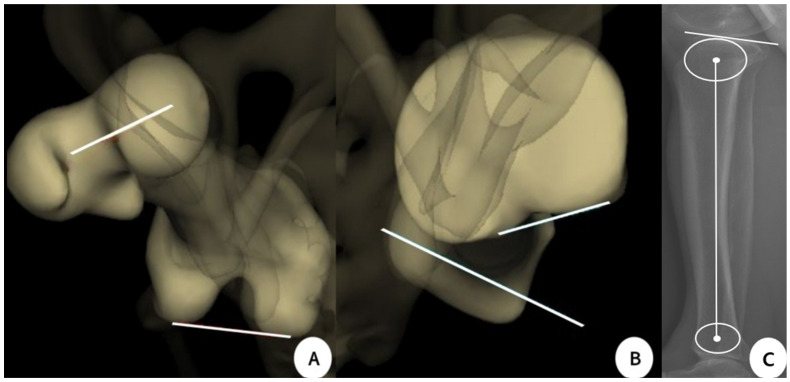
EOS measurements. (**A**) Femoral torsion; (**B**) Tibial torsion; (**C**) Posterior tibial slope on the sagittal plane of the EOS.

**Figure 4:**
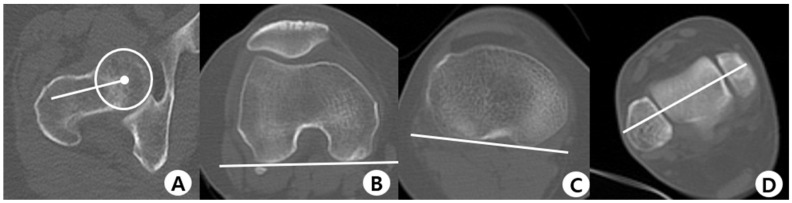
Axial computed tomography. (**A**) Femoral neck axis; (**B**) The line adapted to the posterior contour of the medial and lateral femoral condyles; (**C**) The line adapted to the posterior contour of the proximal tibial head; (**D**) The bimalleolar axis.

**Figure 5:**
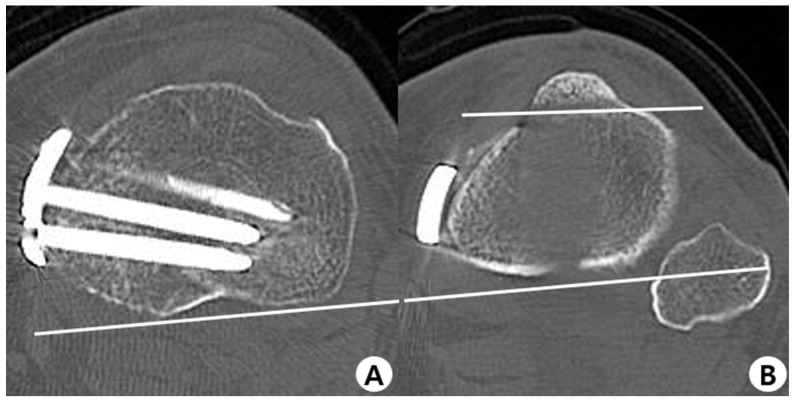
Measurement of the tibial osteotomy angle. (**A**) The line adapted to the posterior tibial plateau; (**B**) The line of the osteotomy at the level of the tibial tuberosity axis.

Images of the EOS and CT measurements are described in Figure 3A,B and Figure 4A–D, respectively. The posterior tibial slope measurements were performed in the sagittal plane. The tibial axis was determined according to the method described by Lipps et al. (Figure 3C). The angle between the proximal posterior tibial axis and the osteotomy axis of the tibial tuberosity was defined as the angle of the tuberosity osteotomy axis (Figure 5A,B) [14,39]. Furthermore, the hip–knee–ankle angle (HKAA), the angle between the lines from the knee joint center to the femoral head center and the ankle joint center, was calculated from the weight-bearing AP long-leg view. When the knee had a varus deformity, the angle had a positive value. In addition, the medial angle between the articular line of the proximal tibia and the anatomical axis of the tibia was defined as the medial proximal tibial angle.

The Western Ontario and McMaster Universities Osteoarthritis Index (WOMAC), Hospital for Special Surgery (HSS) score, and range of motion of the involved knees were used to evaluate functional outcomes in this study. Additionally, patient-reported outcomes were collected twice in the clinic by a single orthopedic surgeon, once preoperatively and once 1 year postoperatively.

### 2.4. Surgical Technique

All operations were performed by a single senior surgeon. A 5 cm anteromedial skin incision was made medial to the tibial tuberosity. Subsequently, the pes anserinus tendon was released, and the superficial medial collateral ligament was exposed and detached with a Cobb elevator. To protect the neurovascular structures of the knee, the Cobb elevator was inserted, and subperiosteal dissection on the posteromedial and posterolateral aspect of the tibia was performed. Diagnostic arthroscopy was performed in all patients for meniscal procedures such as repair or meniscectomy before MOWHTO, and lateral retinacular release was performed after the arthroscopic procedure. Guidewires were inserted approximately 3.5 cm below the medial joint line and directed obliquely toward the tip of the fibula head. Biplane osteotomy was performed at the medial tibial cortex using an oscillating saw. The osteotomes were carefully advanced to approximately 1 cm into the lateral cortex without breakage. The osteotomy site was carefully widened to the planned width using a chisel. When the expected osteotomy width was reached, we checked whether the mechanical axis passed the 62.5% point of the tibial plateau using the cable method. Stabilization of the osteotomy site was achieved using a metal plate with a metal block (Ohtofix, Ohtomedical Co., Ltd., Goyang, Republic of Korea).

### 2.5. Statistical Analysis

The normality of the distribution was confirmed by performing the Kolmogorov–Smirnov test. The rotational alignment and slope of the lower extremity were measured by two orthopedists in CT taken before surgery. The average value of the values measured by two orthopedists was determined as the preoperative rotational alignment and slope values, and the CT images taken after the surgery were also measured in the same way. The change between the values of each variable before and after surgery was calculated. The amount of change in variables of EOS before and after surgery was also calculated. Student’s *t*-tests were then performed to compare changes in femoral torsion, tibial torsion, femorotibial rotation, and posterior tibial slope between the CT and EOS groups. In addition, pre- and postoperative comparisons of functional outcomes, tibial slope, tibial rotation, femorotibial rotation, the HKAA, and the medial proximal tibial angle were performed using paired *t*-tests. Pearson’s correlation coefficients were calculated to investigate the association between the variables and the changes in tibial torsion and posterior tibial slope after MOWHTO in patients with pre- and postoperative CT, regardless of the EOS system. All statistical analyses were performed using SPSS version 20.0 (IBM Corp., Armonk, NY, USA), and statistical significance was set at *p* < 0.05.

## 3. Results

All patients were diagnosed with medial compartment osteoarthritis with varus malalignment. The population consisted of 22 females (73%) and 8 males (27%), with a mean age of 59.9 years at the time of surgery. There were 16 and 14 left and right knees, respectively. Furthermore, the mean preoperative flexion contracture was 4.0 degrees, while the average correction angle calculated using the Dugdale and Noyes method was 8.27 (4.0–15.0 degrees). MOWHTO was combined with medial meniscus repair in 19 knees (63%) and partial medial meniscectomy in 11 knees (37%). Additionally, 2/25 (7%) patients underwent simultaneous MOWHTO and allogeneic mesenchymal stem cell transplantation (Table 1).

The mean differences in femoral torsion between pre- and post-MOWHTO when using CT and the EOS system were 0.17° ± 2.73° and −2.96° ± 9.24°, respectively; however, there were no statistically significant results between these values (*p* = 0.142). In addition, the mean differences between pre- and postoperative tibial torsion when using CT and EOS were −3.59° ± 2.64° and −3.39° ± 7.34°, respectively (*p* = 0.894). Furthermore, the mean difference of the femorotibial rotation when using CT was 2.61° ± 3.63°, while that for the EOS system was 2.46° ± 10.58° (*p* = 0.947). When the posterior tibial slope was measured before and after MOWHTO, the change observed when using the EOS system was 1.07° greater than that in CT, which was not statistically significant (*p* = 0.227) (Table 2).

The clinical and radiological outcomes after MOWHTO are described in Table 3. We observed that the posterior tibial slope increased by 0.82° ± 1.92° after MOWHTO (*p* = 0.035). Additionally, the tibial rotation showed a change of −3.59° ± 2.64° between the pre- and postoperative measurements, which was statistically significant (*p* < 0.001). Similarly, the femorotibial rotation showed a statistically significant change of 2.61° ± 3.63° between the pre- and postoperative measurements (*p* < 0.001). Furthermore, the mean HKAA changed from varus (6.29° ± 2.33°) to valgus (−1.11° ± 2.10°) after MOWTHO, which was statistically significant (*p* < 0.001). In terms of clinical outcomes, the WOMAC scores decreased in all categories after MOWHTO. The total WOMAC and WOMAC pain scores had statistically significant outcomes: *p*-values of 0.008 and 0.007, respectively. The HSS score also increased postoperatively by 8.75 ± 3.58 (*p* < 0.001).

Using Pearson’s correlation analysis in 37 patients, the correlations between the change in tibial torsion and posterior tibial slope (difference between pre- and postoperative values) and each independent variable were assessed (Table 4). No variables showed a significant correlation between the changes in the tibial torsion or posterior tibial slope.

## 4. Discussion

The main finding of this study was that there was no significant difference between the CT and EOS systems when measuring the changes in femoral rotation, femorotibial rotation, tibial rotation, and posterior tibial slope. This suggests that the EOS system developed for evaluating lower extremity alignment may replace CT, the gold standard for measuring lower extremity profiles. Additionally, we confirmed that the distal tibia was internally rotated as a result of MOWHTO; however, there was no significant correlation between the degree of internal rotation and various parameters and functional results.

Since the development of the EOS system, studies comparing the EOS system with CT for evaluating lower extremity alignment have been published. Folinais et al. reported that rotation parameters measured using the EOS system strongly correlated with the values measured by CT. Buck et al. reported that measuring femoral and tibial torsion using 3D models based on the EOS system could replace standard CT measurements in patients with knee osteoarthritis [33,34]. Similarly, Hecker et al. reported that posterior tibial slope measurements using the EOS 3D imaging system were as reliable and reproducible as those obtained using CT [40]. Previous cross-sectional studies have also reported that EOS can replace CT; however, our study is novel in that we examined the differences between the EOS system and CT before and after surgery and found that the EOS system can replace CT for these measurements.

It was reported that the difference between EOS and CT was about 3 degrees on average in the femoral torsion in the study comparing EOS and CT [33,34]. In the femoral torsion values in Table 2, the CT showed the result as anteversion, and the EOS showed the opposite result (CT: 0.17°/EOS system: −2.96°). Research that MOWHTO affects femoral torsion has not been confirmed yet, and it is thought that MOWHTO does not affect the alignment of femoral torsion. Therefore, the different results were thought to be due to the differences in the diagnostic methods, i.e., the CT and EOS systems.

Some studies have reported coronal realignment, unintended rotation of the distal fragment in the axial plane, and increases in the posterior tibial slope occurring after HTO surgery [9,10,14,23]. These unintended deformities can adversely affect the knee and ankle joints and contribute to patellofemoral joint arthritis. Therefore, care should be taken by surgeons when performing HTO. Our study also showed that the distal fragment was internally rotated, and the posterior tibial slope was increased after MOWHTO, as shown in Table 3. Therefore, because realignment of the axial and sagittal planes could occur during surgery, particular care was taken during MOWHTO. However, we cannot rule out the possibility that statistically significant realignment occurred during technical aspects such as plate fixation (performed with the distal bone fragment slightly extended) or as the bone fragments were fixed with reduction forceps to improve the contact of the anterior surface in biplane osteotomy. Notably, Suh et al. reported that the increase in posterior tibial slope was lower in uniplane medial-opening HTO than in biplane HTO, suggesting that the use of biplane MOWHTO in our study may have also affected the increase in posterior tibial slope [41].

Many studies have confirmed the occurrence of realignment after MOWHTO; however, factors that cause greater deformation have not been reported [9,10,14,23,25,42]. Hinterwimme et al. reported that distal tibial rotation was not related to the correction angle; instead, they assumed that rotation of the distal tibia would occur because of the influence of the 3D anatomic complexity of the tibia and the state of peripheral soft tissue, especially the semitendinosus and gracilis tendons [10]. Moreover, Jang et al. reported that a greater opening width and tuberosity osteotomy angle during MOWHTO showed a tendency for greater internal rotation of the distal tibia [14]. Similarly, Kim et al. reported that the distal tibial fragment was externally rotated after MOWHTO, and this degree was related to the opening gap width [42]. In line with these previous studies, we confirmed the relationship between the correction angle, tibial osteotomy angle, and preoperative flexion contracture with the degree of realignment; however, these results were not statistically significant. In addition, we confirmed that the degree of deformation did not affect functional outcomes in the first year after surgery; this result was also not statistically significant (Table 4).

As tibial rotation changes after high tibial osteotomy, several studies have reported that the posterior tibial slope also changes after high tibial surgery. The axis of the osteotomy hinge has been studied as a significant determinant in determining the change in the posterior tibial slope during MOWHTO [13,19,20,22,23,43]. Theoretically, the internally rotated osteotomy hinge axis may decrease the posterior tibial slope, and the externally rotated hinge axis may increase the posterior tibial slope. Wang et al. reported that the posterolateral osteotomy axis increased the posterior tibial slope after MOWHTO more than the lateral osteotomy axis [23]. Claire et al. reported that the distalization–flexion position of the hinge axis also increased the posterior tibial slope in addition to the external rotation of the osteotomy axis [20]. Therefore, MOWHTO should be performed in a way that the axis of osteotomy rotates internally and angulates proximalization–extension simultaneously to prevent changes in the posterior tibial slope, but this is challenging. The surgeon also paid much attention to not changing the posterior tibial slope in this study, but the result showed that the angle increased by an average of about 0.8 degrees after MOWHTO, which was a statistically significant change before and after surgery. Therefore, it is necessary to pay more attention to the determination of the hinge axis of osteotomy, keeping in mind that the posterior tibial slope may change after MOWHTO.

Research using computer simulation is a research method that is more convenient and can obtain faster results than research conducted in a laboratory or on patients. Various in silico studies have also been reported by realizing bones or surgical instruments as images using 3D CT reconstruction in orthopedics [44,45,46,47,48]. Recently, a finite element analysis on the pressure applied to the knee cartilage according to the correction angle after HTO surgery has been published. The study reported that the desired alignment was achieved under a valgus hypercorrection of 4.5° that significantly unloads the medial compartment, loads the lateral compartment, and arrests the progression of osteoarthritis [49]. In addition to this, it is possible to conduct research (using computer stimulation) on how much rotation of the distal tibia occurs and how the slope changes due to the influence of the correction angle, osteotomy axis, opening gap width, and soft tissue. It is also necessary to study the change in pressure applied to the knee joint and ankle joint by considering the change in the mechanical axis due to high tibial osteotomy and the rotation and slope change of the tibia by various factors simultaneously. It will be helpful to find the optimal correction angle and osteotomy axis when performing HTO surgery by considering both the slope and rotation as well as the change in mechanical axis.

An advantage of the EOS system is the reconstruction of 3D data based on biplanar radiographs (Figure 2); therefore, it is regarded as a new diagnostic method for femoral and tibial torsion measurements that can replace preoperative radiographs and CT. Visualization of the spinal geometry is enabled by the 3D reconstruction by the EOS system in the horizontal plane view from above, which provides the surgeon with more information in orthopedic surgery, especially in scoliosis surgery. The EOS system can help patients with multiple abnormalities, especially when they are present simultaneously [50]. In addition, because the radiation exposure of the EOS system is significantly lower than that of X-ray or CT, alignment measurement using EOS imaging is advantageous for children and adolescents, especially patients who need various orthopedic imaging studies [51]. Dietrich et al. reported that the radiation dose is 50% lower in the EOS system than in X-ray imaging for the entire lower limb [52]. CT involves a fairly high radiation dose because of the broad scanning area. In addition, EOS imaging and various parameters can be acquired simultaneously [53].

The EOS system can be used in various ways, including preoperative planning and postoperative alignment evaluation in spine and lower extremity surgery. Orfeuvre et al. published a study using the EOS system to evaluate postoperative nonunion in patients with femur shaft fractures who underwent intramedullary nailing [54]. Peeters et al. evaluated pedicle size using the EOS system in scoliosis patients [55]. Evaluating pelvic tilt and the position of the acetabulum using the EOS system after total hip arthroplasty was studied by Loppini et al. [56].

The EOS system’s 3D model reconstruction requires accurate landmarks of the bone and is influenced by the standing position before taking an image [57]. In this study, the correct standing position was achieved by standing with one foot slightly anterior to the other. If this is not performed, superimposed knees can negatively affect the identification of the anatomical landmarks of the femur and tibia on the lateral image [35]. However, Cho et al. evaluated the reliability of lower extremity alignment measurements using the EOS imaging system as the patient stood with feet placed parallel in an even weight-bearing posture. They confirmed a significant difference in the tibial and femorotibial rotation [58]. Therefore, we used the adjusted standing position in our implementation of the EOS system.

Our study has a few limitations. First, the number of enrolled patients was relatively low and the patient population lacked diversity; however, the study was conducted only with pure data, and it was statistically reasonable. Second, only two orthopedists interpreted the CT results, which can introduce bias. Third, the follow-up period of 1 year was short. However, this study had the advantage of being a prospective study, unlike other previous studies that compared the EOS system and CT, which were retrospective. We also simultaneously compared the EOS system and CT and investigated the postoperative outcomes of MOWHTO.

In conclusion, this study confirmed the possibility that the EOS system could replace CT in measuring changes in several parameters pre- and postoperatively. Furthermore, we confirmed that the distal tibia tended to be internally rotated after MOWHTO; however, we found no significantly related parameters related to deformation caused by MOWHTO.

## Figures and Tables

**Table 1:** Patient demographics.

Parameters	Knees (*n* = 30)
Sex	
Male	8 (27)
Female	22 (73)
Age (years)	59.9
Height (cm)	159.8
Weight (kg)	67.7
Body mass index (kg/m^2^)	26.41
Side	
Left	16 (53)
Right	14 (47)
Flexion contracture (°)	4.0
Correction angle (°)	8.25 (4–15)
Concomitant surgery	
Medial meniscus repair	19 (63)
Partial medial meniscectomy	11 (37)
Allogenic mesenchymal stem cell transplantation	2 (7)

Data are presented as *n* (%) or mean (range).

**Table 2:** Measurement of the lower extremity rotation and posterior tibial slope using the EOS system and CT.

	CT			EOS System			
	Before MOWHTO (SD)	After MOW1HTO (SD)	Change (SD)	Before MOWHTO (SD)	After MOWHTO (SD)	Change (SD)	*p*-Value
Femoral torsion (°)	14.42 (5.87)	14.59 (6.47)	0.17 (2.73)	17.39 (7.28)	14.96 (9.50)	−2.96 (9.24)	0.142
Tibial torsion (°)	25.16 (6.86)	21.57 (5.21)	−3.59 (2.64)	26.29 (7.97)	22.89 (6.37)	−3.39 (7.34)	0.894
Femorotibial rotation (°)	0.87 (3.66)	3.48 (5.14)	2.61 (3.63)	−1.64 (7.50)	0.82 (9.49)	2.46 (10.58)	0.947
Posterior tibial slope (°)	12.10 (3.33)	12.92 (3.66)	0.82(1.91)	12.75 (4.26)	14.64 (4.53)	1.89 (2.10)	0.227

CT, computed tomography; SD, standard deviation; MOWHTO, medial open-wedge high tibial osteotomy.

**Table 3:** Clinical and radiologic outcomes after MOWHTO.

	Preop. (SD)	Postop. (SD)	Change (SD)	*p*-Value
Radiologic outcomes				
Tibial slope (°)	12.10 (3.33)	12.92 (3.66)	0.82 (1.92)	0.035 *
Tibial rotation (°)	25.16 (6.86)	21.57 (5.21)	−3.59 (2.64)	<0.001 *
Femorotibial rotation (°)	0.87 (3.66)	3.48 (5.14)	2.61 (3.63)	<0.001 *
HKAA (°)	6.29 (2.33)	−1.11 (2.10)	−7.39 (2.74)	<0.001 *
MPTA (°)	84.54 (2.15)	89.89 (3.29)	5.36 (3.88)	<0.001 *
Clinical outcomes (POD 1Y)				
ROM (◦)	131.64 (7.68)	132.46 (7.39)	0.82 (2.73)	0.129
WOMAC score				
Total	44.50 (8.68)	41.21 (4.97)	−3.29 (5.99)	0.008 *
Pain	9.04 (3.92)	7.54 (2.44)	−1.50 (2.69)	0.007 *
Function	30.21 (6.75)	28.61 (4.32)	−1.61 (4.93)	0.102
Stiffness	5.21 (1.15)	5.07 (1.22)	−0.14 (0.91)	0.424
HSS score	71.18 (6.37)	79.93 (7.13)	8.75 (3.58)	<0.001 *

SD, standard deviation; MOWHTO, medial open-wedge high tibial osteotomy; POD, postoperative duration; HKAA, hip–knee–ankle angle; MPTA, medial proximal tibial angle; Y, year; ROM, range of motion; WOMAC, Western Ontario and McMaster Universities Osteoarthritis Index; HSS, Hospital for Special Surgery; * Statistical significance was set at *p* < 0.05.

**Table 4:** Correlations of the changes in tibial torsion and posterior slope with other parameters.

	Change in Tibial Torsion	*p*	Change in Posterior Tibial Slope	*p*
	Pearson’s Correlation Coefficient (r)		Pearson’s Correlation Coefficient (r)	
Correction angle (°)	0.150	0.377	0.186	0.269
TOA (°)	−0.060	0.730	0.027	0.878
Flexion contracture (°)	−0.205	0.224	−0.125	0.462
Change of tibial torsion	1.0		0.000	0.998
Change of posterior tibial slope	0.000	0.998	1.0	
Change of clinical outcomes (POD 1Y)				
ROM (°)	0.173	0.306	−0.036	0.834
WOMAC score				
Total	0.104	0.538	−0.130	0.445
Pain	0.110	0.515	−0.156	0.358
Function	0.05	0.769	−0.056	0.742
Stiffness	0.172	0.309	−0.050	0.770
HSS score	−0.113	0.505	0.250	0.135

TOA, tuberosity osteotomy angle; POD, postoperative duration; ROM, range of motion; WOMAC, Western Ontario and McMaster Universities Osteoarthritis Index; HSS, Hospital for Special Surgery.

## Data Availability

Deidentified data are available on reasonable request from the corresponding author.
